# LncRNA Gm20063 alleviates cognitive impairment in APPswe/PS1dE9 mice

**DOI:** 10.1002/alz.085039

**Published:** 2025-01-03

**Authors:** Xiaolei Zhu, Zhen Lan, Yun Xu

**Affiliations:** ^1^ Affiliated Drum Tower Hospital of Nanjing University Medical School, Nanjing China; ^2^ Affiliated Drum Tower Hospital of Nanjing University Medical School, Nanjing, Jiangsu China

## Abstract

**Background:**

Compelling evidence has shown that long non‐coding RNAs (lncRNAs) contribute to Alzheimer’s disease (AD) pathogenesis including β‐amyloid plaque deposition (Aβ) and intracellular neurofibrillary tangles. In this study, we aimed to investigate the critical role of lncRNA Gm20063 in AD.

**Method:**

Six‐month‐old male APP/PS1 transgenic mice and wild type (WT) C57BL/6 (B6) littermates were obtained from the Nanjing University Animal Model Research Center. AAV mediated over‐expression or knockdown of Gm20063 in hippocampus neurons though stereotactic technique. Behavioral tests were performed to evaluate the cognitive and memory function of APP/PS1. Electrophysiological tests, Golgi staining and Western blotting was applied to determine the synaptic plasticity. The potential targets of Gm20063 were identified by mass spectrometry (MS), RNA‐binding protein immunoprecipitation (RIP) and RNA in situ hybridization (RNA scope).

**Result:**

The expression of Gm20063 was significantly increased in the hippocampus of APP/PS1 mice. Overexpression of Gm20063 ameliorated cognitive decline, reduced Aβ deposition, enhanced synaptic plasticity and Aβ phagocytized by microglia in APP/PS1. In addition, knockdown of Gm20063 accelerated the memory deficits and worsened the AD pahogenesis in APP/PS1 mice. Furthermore, Gm20063 interacted with TDP‐43 in the hippocampus, and regulated the post‐translational modifications of TDP‐43.

**Conclusion:**

Gm20063 attenuated cognitive decline in AD mice, and targeting Gm20063 might be an effective therapy for AD treatment.

